# The effects of TORC signal interference on lipogenesis in the oleaginous yeast *Trichosporon oleaginosus*

**DOI:** 10.1186/s12896-017-0348-3

**Published:** 2017-03-07

**Authors:** Felix Bracharz, Veronika Redai, Kathrin Bach, Farah Qoura, Thomas Brück

**Affiliations:** Industrial Biocatalysis Group, Technische Universität München, Lichtenbergstraße 4, 85748 Garching, Germany

**Keywords:** *Trichosporon oleaginosus*, Rapamycin, TORC, Single cell oil, Oil yeast

## Abstract

**Background:**

Oleaginous organisms are a promising, renewable source of single cell oil. Lipid accumulation is mainly induced by limitation of nutrients such as nitrogen, phosphorus or sulfur. The oleaginous yeast *Trichosporon oleaginosus* accumulates up to 70% w/w lipid under nitrogen stress, while cultivation in non-limiting media only yields 9% w/w lipid. Uncoupling growth from lipid accumulation is key for the industrial process applicability of oleaginous yeasts. This study evaluates the effects of rapamycin on TOR specific signaling pathways associated with lipogenesis in *Trichosporon oleaginosus* for the first time.

**Results:**

Supplementation of rapamycin to nutrient rich cultivation medium led to an increase in lipid yield of up to 38% g/L. This effect plateaued at 40 μM rapamycin. Interestingly, the fatty acid spectrum resembled that observed with cultivation under nitrogen limitation. Significant changes in growth characteristics included a 19% increase in maximum cell density and a 12% higher maximum growth rate. *T. oleaginosus* only has one Tor gene much like the oleaginous yeast *Rhodosporidium toruloides*. Consequently, we analyzed the effect of rapamycin on *T. oleaginosus* specific TORC signaling using bioinformatic methodologies.

**Conclusions:**

We confirm, that target of rapamycin complex 1 (TORC1) is involved in control of lipid production and cell proliferation in *T. oleaginosus* and present a homology based signaling network. Signaling of lipid induction by TORC1 and response to carbon depletion to this complex appear to be conserved, whereas response to nitrogen limitation and autophagy are not. This work serves as a basis for further investigation regarding the control and induction of lipid accumulation in oil yeasts.

**Electronic supplementary material:**

The online version of this article (doi:10.1186/s12896-017-0348-3) contains supplementary material, which is available to authorized users.

## Background

Production volumes of bio-based lipids have experienced a 65% increase over the last decade due to increasing demands by food, chemical- and pharmaceutical industries [1, 2]. Especially the application of natural oils for biofuels, oleochemicals and bioactive substances is expanding rapidly [3]. However, the application of plant oils for non-food use accelerates land use and climate change, which in turn negatively impacts on biodiversity. Further, the majority of pharmaceutically active high value lipids such as long chain polyunsaturated fatty acids are still sourced from fish and crustaceans, which negatively affects sensitive marine ecosystems.

Microbial oils have been designated as a sustainable alternative to plant and animal based lipids. In particular, oleaginous yeast, which can accumulate between 20 and 70% w/w lipids [4, 5] have gained increasing interest as providers for sustainable oleochemical building blocks in the biofuel, lubricant, food and cosmetics industry. However, induction of lipogenesis in microorganisms is linked to nutrient restriction (N or P), which results in significant reductions in cell growth. In order to design an economically relevant continuous production process that provides for high lipid and biomass formation the metabolic uncoupling of cell growth from intracellular lipid accumulation is a prerequisite. This aim however requires in depth knowledge of the cells regulatory mechanisms involved in lipogenesis.

The target of rapamycin complexes (TORCs) are central, highly conserved regulators in the control of cell proliferation, sexual development, cell skeleton organization, lipogenesis and other essential functions. TORCs draw their name from the TORC1 inhibiting heterocyclic makrolid rapamycin, derived from *Streptomyces hygroscopicus*. The complexes consist of different components depending on the organism. The key component is Tor1, a phosphatidylinositol 3 kinase-like serine/threonine kinase (mTOR in mammals).

Whereas some involvement of TORCs, such as TORC1 activation of translation and transcription are conserved from yeast over plants to mammals [6], other downstream processes are vastly different depending on the organism. It is reported that the signaling network plays a crucial role in the control of lipid homeostasis [7, 8]. Understanding the regulatory system of lipid accumulation would therefore contribute to the ability for targeted, genetic modification of single cell oil production strains.

Recently, the effects of rapamycin on the lipid accumulating microalgae *Euglena gracilis* in comparison to model algae *Chlamydomonas reinhardtii* and *Cyanidioschyzon merolae* were characterized [9]. Lipid content was significantly increased when the algae were exposed to minor concentrations of rapamycin. By contrast, higher rapamycin concentrations resulted in growth inhibition. While the effect of rapamycin has been well described in model yeasts, it has as of now not been evaluated in non-conventional oil forming yeasts strains. Therefore, this study evaluates the significance of TORC signaling pathways on lipogenesis in oleaginous yeast for the first time.

## Methods

### Strains and media

Wild type *Trichosporon oleaginosus* ATCC 20509 (DSM-11815), obtained from the “Deutsche Sammlung von Mikroorganismen und Zellkulturen” (DMSZ) (Braunschweig, Germany) was used for all experiments. Cultivation was done in YPD medium (glucose, 20 g/L; tryptone, 20 g/L; yeast extract, 10 g/L), nitrogen limitation medium [10] (glucose, 30 g/L; yeast extract, 0.5 g/L; (NH4)2SO4, 0.3 g/L; MgSO_4_•7H_2_O, 1.5 g/L; KH_2_PO_4_, 2.4 g/L; Na_2_HPO_4_ 0.91 g/L; CaCl_2_•H_2_O, 0.22 g/L; ZnSO_4_•7H_2_O, 0.55 μg/L; MnCl_2_•4H_2_O, 22.4 μg/L; CuSO_4_•5H_2_O, 25 μg/L; FeSO_4_•7H_2_O, 25 μg/L, pH 6.1). Rapamycin (Tecoland, CA, USA) was solved in DMSO (10 mM stock) and added directly to the media in different concentrations after the inoculation.

### Cultivation conditions

*T. oleaginosus* was cultivated as triplicate in yeast extract peptone dextrose medium (YPD) with different concentrations of rapamycin solution for 7 days at 28 °C in 500 mL baffled shake flasks. Cultivation was carried out in 100 mL YPD and nitrogen limitation medium (MNM) with glucose. Cells from an overnight culture grown in YPD medium under the same cultivation conditions were washed in ddH_2_O and used to inoculate all cultivations at OD_600_ = 0.5. Where applicable, rapamycin was added 8 h after inoculation and adjusted to varying concentrations. 6 mL samples were taken daily for analysis of cell-dry weight, lipid content and fatty acid distribution.

### Biomass and lipid determination

Determination of cellular dry weight occurred by pelleting 2 mL samples (12,000 g for 10 min), washing cells with ddH_2_O and freeze drying at −80 °C for 24 h in pre-weighed microtubes. Cellular total lipid was obtained by extraction with chloroform and methanol by Folch et al. [11] (adapted). Cell pellets from 12 mL culture were washed with ddH_2_O twice and disrupted four times by french press (EmulsiFlex®-B15, Avestin) at 2400 bar. A triplicate of 4 mL cell lysate was transferred to glass vials with screw caps and mixed with 6 mL of Folch reagent (2:1 chloroform/methanol) each. After extraction by shaking at 900 rpm and room temperature for 1 h, 1 mL 0.9% NaCl was added to aid phase separation. Samples were vortexed, centrifuged at 1000 g and the chloroform phase was transferred to pre-weighed glass vials. After evaporation of the solvent und a constant stream of dried nitrogen, vials were weighed and lipid content was calculated per dry weight in % g/g.

### Analysis of fatty acid composition

Triplicates of 2 mL culture were pelleted by centrifugation, washed with ddH_2_O and freeze dried at −80 °C. Between 10 and 20 mg were used for the fatty acid analysis. Fatty acid methyl esters (FAMES) were obtained by direct conversion of cell biomass by methanol transesterification [12].

FAMEs were analyzed on a GC-2025 gas chromatograph from Shimadzu (Nakagyo-ku, Kyōto, Japan) with flame ionisation detector and an AOC-20i auto injector (Shimadzu). 1 μl sample was applied onto a ZB-WAX column (30 m, 0.32 mm ID; 0.25 μm df; Phenomenex (Torrance, CA, USA)) with an initial column temperature of 150 °C (maintained for 1 min). A temperature gradient was applied from 150–240 °C (5 °C/min), followed by 6 min maintenance at 240 °C. Fatty acids were identified according to retention times of authentic standards.

### Nile red assay

Semi-quantitative estimation of intracellular lipids was done by Nile Red staining according to Sitepu et al. [13]. 200 μL of culture sample was adjusted to OD_600_ = 1 and transferred to MaxiSorp F96 plates (Thermo Scientific Waltham, MA, USA) as 5 replicates. After addition of 25 μL DMSO, the blank measurement was taken. Subsequently, 25 μL of Nile Red staining solution (0.1 mg/mL in DMSO) were added and Nile Red fluorescence intensity was measured at 590 nm (excitation 530 nm) on an EnSpire 2 plate reader from Perkin Elmer (Waltham, MA, USA). Lipids were estimated by correcting FI (fluorescence intensity) by OD_600_.

### Online OD measurements

Online OD measurements were conducted by measuring real-time backscatter at 525 nm with a Cell Growth Quantifier (Aquila biolabs - Baesweiler) using 100 mL YPD in 250 mL shake flask without baffles for 72 h. Rapamycin concentration was adjusted to 5 μM 8 h after inoculation. The backscatter signal was calibrated with a manual 2 point OD_600_ measurement by an HP 8453 photometer.

### Statistical analysis

Statistical analysis and data visualization was done in R [14]. Data were fitted to a Richards’ growth curve using the grofit package [15]. All error bars show standard deviations. Stars show significance with *p* = 0.05 in comparison to untreated cultures.

### Assembly of TORC-network

Previously published genomic and transcriptomic data of *T. oleaginosus* [16] were searched for TORC homologues using sequences from *Schizosaccharomyces pombe* and *Saccharomyces cerevisiae, Candida curvata* and *Cryptococcus neoformans*.

## Results and discussion

### Effect of rapamycin on nile red fluorescence

**Fig. 1:**
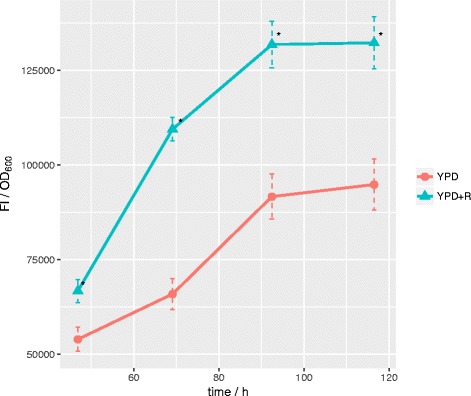
Effect of rapamycin on nile red fluorescence: *T. oleaginosus* grown in YPD without (YPD) and with 20 μM rapamycin (YPD + R) supplementation. At different time points, nile red fluorescence (FI) and OD_600_ were measured. The ratio between the latter is plotted on the y axis and is a semiquantitative indicator of lipid content. Stars show significance at *p* = 0.05 and error bars show standard deviation

Cultivation of *T. oleaginosus* in full YPD medium does not lead to nutrient limitation and is associated with a low accumulation of intracellular lipids. Supplementation of this medium with rapamycin (20 μM, [9]) after 8 h cultivation time resulted in significantly higher nile red fluorescence (corrected for OD) signal indicating an increased lipid production (Fig. 1). The increase of 66% FI/OD at 72 h subsequently decreased to 44% (92 h) and 40% after 116 h respectively. During cultivation, no decrease in OD was observed, indicating that the cell growth was not affected by the applied rapamycin concentrations.

It is reported that Nile Red is a semiquantitative lipid stain [17], as its specific fluorescent signal is dependent on the fatty acid profile, the type of lipid (phospho-, triacylglyceride or steran) and the protein content within intracellular lipid bodies.

### Effect of rapamycin on lipid accumulation

To confirm an increase in lipid yield, a gravimetric biomass and lipid determination was conducted using cells grown in cultures containing between 0 and 40 μM rapamycin. The addition of 40 μM rapamycin resulted in a maximal lipid increase of 38% compared to controls. Moreover, at 5 μM rapamycin the total biomass production is significantly increased. This data contrasts reports on the effects of rapamycin on algae and bacterial growth, where supplementation of the compound resulted in decreased biomass formation in line with its established cell cycle inhibition effects [8, 18].

**Fig. 2:**
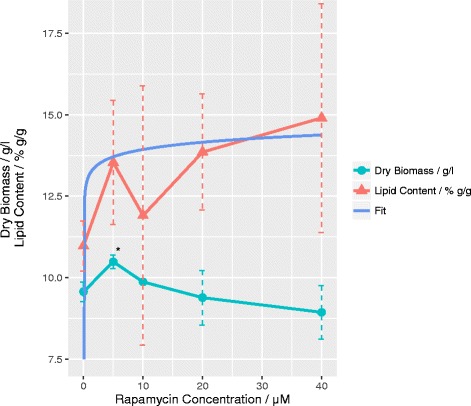
Effect of rapamycin on lipid content and biomass: Total lipid content (*triangle*) and dry biomass (*circle*) of *T. oleaginosus* after 72 h of cultivation in YPD are measured with different concentrations of rapamycin supplementation between 0 and 40 μM. Error bars show standard deviation and the star shows significance at *p* = 0.05 in comparison to culture without rapamycin supplementation. The *blue line* shows a robust logarithmic fit of the lipid content in dependence of rapamycin concentration as described in Additional file 1

Due to the expected logarithmic dependency (Additional file 1), effects of rapamycin on lipid content (Fig. 2) saturate at low concentrations. To confirm the effect of rapamycin on intracellular lipogenesis, we applied a one tailed Welch’s t-test between samples in the absence and presence of rapamycin. The null hypothesis of both sample sets being of the same distribution is rejected with *p* = 0.003 and a confidence interval of 1.4–5% g/g increase in the absence and presence of rapamycin.

### Impact of rapamycin on *T. oleaginosus* growth kinetics

**Fig. 3:**
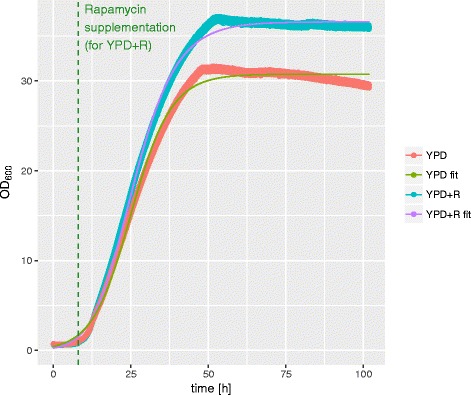
Online-OD measurement: Growth curves of *T. oleaginosus* without (YPD) and with 5 μM rapamycin (YPD + R) obtained by measuring backscatter with an online OD system. *Thin lines* (YPD fit, YPD + R fit) show fit of Richards’ growth curves

As our initial data suggested that at 5 μM rapamycin (at t: 8 h cultivation time) cell growth was enhanced, we applied a real-time backscatter measurement in order to compare cellular growth in the absence and presence of rapamycin (Fig. 3). Cell growth could be sufficiently described by a fit to a Richards’ curve [19], which allowed extraction of μ_max_ (maximum growth rate), lambda (lag phase duration) and A (maximum OD_600_) values (Table 1).

**Table 1:** Comparison of growth curve parameters extracted from Richards’ fit: Maximum growth rate (μ_max_), lag phase (λ) and maximum cell density (A) of *T. oleaginosus* cultivated in YPD with and without rapamycin

	YPD	YPD + 5 μM rapamycin	Change / %
μ_max_ / OD_600_/h	1.2068 ± 1.779*10^−3^	1.349 ± 1.362*10^−3^	11.79 ± 2.6*10^−3^
λ / min	12.433 ± 23.104*10^−3^	12.151 ± 5.776*10^−3^	−2.27 ± 3.13*10^−3^
A /OD_600_	30.754 ± 7.421*10^−3^	36.578 ± 6.73*10^−3^	18.93 ± 0.46*10^−3^

All values are given with standard deviations

Interestingly, we observed significant differences in the growth parameters of each culture. For the rapamycin treated culture, the maximum growth rate μ_max_ (12% increase) and the maximum optical density (19% increase) were elevated compared to controls.

This translates to a change in the maximum growth rate from a nominal 1.2 to 1.35 OD_600_/h and an increase in the maximum cell density from OD 30.8 (61 g/L) to OD 36.6 (71 g/L) respectively. Hence, in the presence of 5 μM rapamycin the cell density increased by 19% concomitantly with a 25% lipid increase. These cumulative values translate to a 49% improved space time yield compared to controls in the absence of rapamycin.

The improved growth rate may be attributed to the upregulation of pathways relating to alternative nitrogen sources. A simultaneous assimilation of many different nitrogen sources could be advantageous in a high nutrient environment such as YPD. Furthermore, TORCs are known to affect cell cycle progression. Shortening of the G2 phase could lead to an increased growth rate, while sacrificing replication fidelity and long term offspring survival.

### Fatty acid profile

**Fig. 4:**
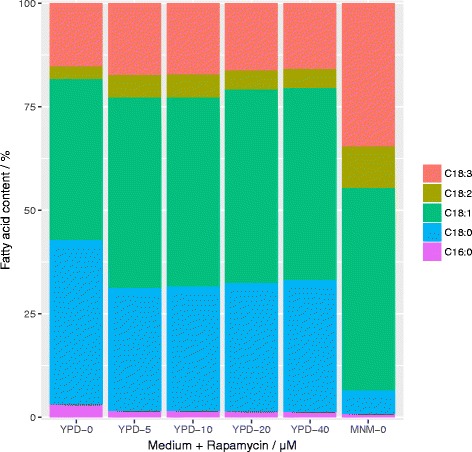
Effect of rapamycin on fatty acid content: Profile of the main fatty acids of *T. oleaginosus* after 72 h of cultivation in Minimal N Medium (MNM-0) or YPD with different rapamycin concentrations (YPD-0 – YPD-40)

Supplementation of rapamycin caused a non-concentration dependent shift in fatty acid spectrum (Fig. 4). Under these conditions, a major decrease of C18:0 in favor of C18:1 fatty acids was observed. Additionally, a minor decrease of C16:0 and a minor increase in C18:3 could be detected. These changes resemble the fatty acid profile obtained by cultivation in nitrogen limiting medium, thereby supporting the notion that rapamycin is at least partially simulating a low nutrient environment to the cells regulatory system. Fatty acid spectra show, that the effect of rapamycin saturates at comparatively low concentrations, confirming the fit in Fig. 2.

In *Euglena gracilis* addition of rapamycin led to an increase in lipid amount but almost no change in fatty acid profile was reported [9]. The reason for this appears to be the different mechanism of rapamycin response between this algae and previously identified yeasts or animal cells [9]. This observation in this study motivated us to use a bioinformatics approach to investigate the effects of rapamycin on the Trichosporon cell signaling network and lipogenesis.

### TORC-network

**Fig. 5:**
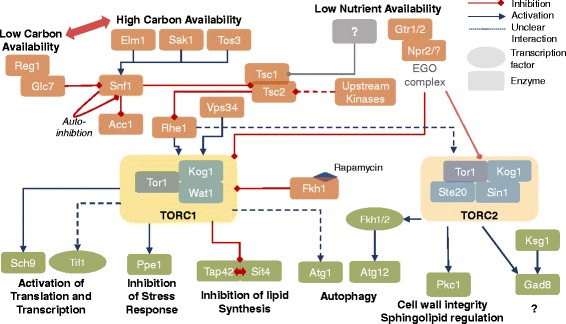
TORC signaling network in *T. oleaginosus:* Proposed signaling network surrounding TORC in *T. oleaginosus* inferred by homology and assembled using data obtained from *S. pombe* and *S. cerevisiae*

A homology-based TORC signaling network, including upstream and downstream elements (Fig. 5), was assembled. A table of all proposed pathway components can be found in Additional file 2.

The SNF1/AMPK pathway is highly conserved from yeast to mammals, and homologues of its main components could also be detected in *T. oleaginosus*. These homologues termed Elm1, Sak1 and Tos3 are kinases which phosphorylate and activate Snf1 (orthologue to the mammalian AMPK), which in turn is a central regulator required for energy homeostasis. In *S. cerevisiae* Snf1 is mainly responsible for adaption to a glucose limiting environment [20]. Inactivation of Snf1 is caused by dephosphorylation initiated by the Reg1/Glc7 complex [20]. In a low carbon environment, phosphorylated Snf is translocated to the nucleus, where it phosphorylates the transcriptional activator Sip4. Subsequently, Sip4 activates the transcription of glucose-repressed genes [20]. This process is conserved in ascomycetes *S. cerevisiae* and *S. pombe* alike. Interestingly, activated Snf1 also inhibits Acc1p, which is responsible for catalysis of acetyl-CoA to malonyl-CoA, a main precursor for the production of fatty acids [21]. This indicates a direct link between metabolic signaling under nutrient stress conditions and the regulation of cellular lipid biosynthesis.

Furthermore, Snf1 is reported to inhibit the Tsc1/Tsc2 complex in model yeasts and in mammals. Moreover, the Tsc1/Tsc2 complex further integrates signals from other pathways, including the MAPK/ERK pathway [22], cytokines, hypoxia signals and Wnt signaling respectively. More recently, it has been suggested that in yeast, involvement of other factors, especially in reaction to low nutrient content is feasible [23]. However, a direct inhibition of TORC1 by Vps34, an essential gene which channels amino acid availability to the Tsc1/Tsc2 complex, has been reported in yeasts and mammals [22, 24].

More specifically, TORC1 itself receives inputs from RheB over the Tsc1/2 axis and directly from the EGO complex. In model yeasts this EGO is composed of the kinases Gtr1/2 and Npr2/3 respectively. However in *T. oleaginosus*, we could not detect homologous of the Npr3 or Ego1-Ego3 complexes, which indicates that the absence of amino acids in the medium are not sensed via the EGO involved signaling. Inhibition of the TORC1 complex by rapamycin occurs via initial formation of an Fkh1 protein-rapamycin complex (mammalian homologue Fkbp12), which then binds to TORC1. This mechanism is highly conserved throughout the microbial and animal kingdom. Expectantly, *T. oleaginosus* Tor1 contained the characteristic rapamycin binding motif [25]. In nitrogen limitied media, the addition of rapamycin has no effect on *T. oleaginosus* biomass formation, growth kinetics and intracellular lipid content. This indicates that TORC1 may already be blocked under nutrient limiting cultivation conditions. Consequently, rapamycin addition would have no effects on biomass or lipid formation in the non-conventional yeast *T. oleaginosus*.

In consensus with data from model yeasts, we could identify homologues components of the TORC1 complex, namely Tor1 (with strong similarity to Tor2 of *S. pombe*), Kog1 and Wat1 respectively. Wat1 is a scaffold protein facilitating the connection between Tor1 and downstream substrates, like Ppe1 and Sch9 [26]. Analogous to model yeasts and mammals the Wat1 protein in *T. oleaginosus* mainly contains 7 WD40 repeats. Specifically in mice, Wat1 was required for TORC2 but not TORC1 activity. Most interestingly, we could not detect any Tco89 homologue in *T. oleaginosus*, which indicates that a component of the TORC complexes found in model yeast *S. cerevisiae* is absent. Therefore, the TORC1 complex of *T. oleaginosus* more closely resembles the situation reported for *S. pombe* or the oleaginous yeast *R. toruloides*. Indeed, this resemblances is confirmed by the absence of a second Tor gene for TORC2. Furthermore, we could not identify any Avo2 or Bit61 homologues in *T. oleaginosus*, both of which are non-functional TORC2-binding structures [27]. Transcripts for the TORC2 component Sin4 could be identified in different splicing isoform, which is consistent with previous findings in *S.cerevisiae* [24]. Notably, in *S. cerevisiae*, mutation of Sin4 leads to rapamycin resistance [28]. In analogy, the presence of Sin4 isoforms detected in *T. oleaginosus* could render the TORC2 complex resistant to rapamycin.

Downstream of primary TORC effects, we could identify significant differences between signals in model yeasts and *T. oleaginosus* affecting autophagy. Particularly, genes essential to the autophagy signaling pathway, namely Atg13, Atg17, Atg31, Atg29 could not found in *T. oleaginosus*. In this respect, the *T. oleaginosus* system may resemble the regulatory system of *Drosophila melanogaster*, in which hyperphosphorylated Atg1 in conjunction with Atg13 are sufficient to inhibit autophagy. In the model yeast S*. cerevisiae* the autophagy signaling is by far more complex and therefore may not apply to *T. oleaginosus* [29]. Further, individual autophagy related homologues (Atg5, Atg6, Atg16) were found, but the actual signaling pathway appears to differ significantly from other yeast systems.

The other main signaling pathways for regulation of lipid biosynthesis, transcriptional and translational initiation appear to be conserved with high similarity.

In *S. cerevisiae*, TORC1 inhibition liberates Tap42 and Sit4 from being bound to each other. This in turn activates the downstream transcription factors, Gat1 and Gln3. After Gat1 and Gln3 transport to the nucleus, these transcription factors induce the accumulation of lipids. Our bioinformatics analysis indicates that a similar mechanism is likely for *T. oleaginosus*. The highly conserved Sch9 is homologous to the mammalian S6K, which is responsible for activation of ribosomal protein S6 and therefore directly controls translation. By contrast, no homologue to Gaf1, which in *S. pombe* is central for the response to nitrogen stress [30], could be identified. The absence of Gaf1 therefore may modulate the cell cycle in *T. oleaginosus*. Nonetheless, Ppe1 homologue a kinase acting within the *S. pombe* stress response which also affects the cell cycle [31] could be identified.

TORC2 activation, especially in yeast, remains elusive. In *S. pombe*, interaction with Rhe1 is confirmed [32]. Furthermore, it was reported in mammals, that activation can be achieved by growth factors (PI3K axis). Furthermore, ribosomal association of the complex suggests its activation by nutrients. Especially the latter is also likely for yeast [33], considering the effect on growth in *S. pombe* [34]. In *T. oleaginosus* however, little can be reported about the effects of downstream TORC2 elements due to the absence of detailed cell biology studies.

Active TORC2 activates Fkh1/2 in *S. cerevisiae* (FOXO genes in mammals), which affects autophagy related genes, life span and stress response [27, 35, 36]. TORC2 as well as the highly conserved kinase Ksg1 activate Gad8 by phosphorylation [37]. The subsequent cellular effects strongly depend on the organism. For *S. cerevisiae*, this impacts on actin organization and cell wall synthesis, whereas for *C. elegans* mainly lipid metabolism and growth are affected. For the related and well described yeast, *S. pombe*, changes in amino acid uptake and general changes in stress response are described [27]. Lack of Rho/Rac homologues indicates a strong difference in the regulation of actin organization from *S. pombe.*

A confirmed element of TORC2 is Pkc activation, as was described for the closely related and pathogenic *Cryptococcus curvatus*. Pkc itself is involved in regulation of spingholipid biosynthesis, which impacts the structural integrity of the cell wall [38]. Most recently, the GATA transcription factor Gaf1 was reported to be responsible for sexual development in yeast and upregulation of amino acid transporters [30]. It is activated as response to nitrogen stress about 10 to 120 min after the onset of nitrogen stress. Therefore it can be hypothesized that it is part of a first, reversible response to nutrient stress. Persisting lack of nitrogen would then trigger the second, delayed phase which includes elevated mating in *S.pombe*.

Two factors indicate, that the observed effects are not due to rapamycin involvement with TORC2: Rapamycin resistance of TORC2 could be structurally substantiated by Avo3 (Ste20), which wraps around the Fkpb-binding domain of Tor1/2 [39]. Prolonged exposure of certain mammalian cell types to rapamycin showed inhibited assembly of TORC2 [40], however this was not observed in unicellular organisms [27] and therefore appears unlikely for *T. oleaginosus*. Secondly, no obvious differences in cell morphology were observed using microscopy (Additional file 3) and FACS (data not shown), indicating that for *T. oleaginosus*, rapamycin does not impact on cytoskeleton and actin organization, which are commonly affected by TORC2.

## Conclusions

For the first time, lipogenesis could be induced in an oleaginous yeast without compromising on growth, resulting in a 1.4 fold increase in total lipid yield. We observed an increase in growth as well as lipid content in the absence of nutrient limitation, using YPD as model substrate with high nutrient content. TORC1 in *Trichosporon oleaginosus* can be inhibited by rapamycin, impacting on growth characteristics and lipid accumulation. However, considering a lack of reduction in growth and comparatively minor increases in lipid accumulation, inactivation of TORC1 is not sufficient to induce a cell state resembling nitrogen starvation.

It is possible, that *T. oleaginosus* either relies on TORC2 inhibition, requires additive signals of both complexes or employs another unknown pathway for full activation of nitrogen stress response and associated lipid accumulation. However, Torc2 regulation of lipid synthesis and its strong involvement in the upregulation of aminoacid transporters [27], one of the defining features of lipid accumulation in *T. oleaginosus*, make TORC2 involvement in nutrient limitation response in this yeast likely.

Proteomic and transcriptomic approaches are excellent tools for elucidating how rapamycin impacts on *T. oleaginosus* physiology. Comparing these data with previously obtained information about transcriptomic changes [16] in the presence of nitrogen stress will allow for pinpointing more clearly the relevance of TORC1 for lipid accumulation. Using a metabolomic strategy is a promising approach for a more in-depth study of key intermediates, such as glutamine and glutamate as components of central nitrogen metabolism.

## Additional files

Additional file 1: Trend analysis: Analytical plots of robust regression using M-estimation of yeast lipid content in dependence of rapamycin concentration in cultivation medium (Fig. 2). Data points 9 and 7, both of which outliers causing deviation at [rapamycin] = 10 were excluded due to high Cook’s distance. The resulting fit was plotted in Fig. 2 and was based on the following formula: Lipid content = 13.1996 + 0.3197*log ([Rapamycin]). (DOCX 60 kb)
Additional file 2: Table of *T. oleaginosus* homologues in TORC signaling network: Abbreviations: *Trichosporon oleaginosus*, TO; *Schizosaccharomyces pombe*, SP; Nitrogen Catabolite Repression, NCR; *Saccharomyces cerevisiae*, SC; *Dictyostelium discodeum*, DD. (DOCX 38 kb)
Additional file 3: Fluorescence microscopy: 200 μL *T. oleaginosus* cells grown for 72 h in YPD with and without 5 μM rapamycin supplementation were pelleted, washed with ddH_2_O and resuspended in the same amount of water. 25 μL DMSO and 25 μL nile red (50 mg/ml) in DMSO were added and incubated in darkness for 10 min. Images were taken on a Zeiss Axio Lab A1 with an Axio Cam ICm1 (Oberkochen, Germany). Fluorescence was measured with a 525/25 filter with an exposure time of 500 ms. (DOCX 154 kb)

## Abbreviations

FACS: Fluorescence activated cell sorting
FI: Fluorescence intensity
MNM: Minimal nitrogen medium
N: Nitrogen
OD: Optical density
P: Phosphorus
S: Sulfur
TORC: Target of rapamycin complex
YPD: Yeast extract pepton dextrose medium
