# Supplementary material for: The effects of TORC signal interference on lipogenesis in the oleaginous yeast Trichosporon oleaginosus
Source: BMC Biotechnol. 2017 Mar 7;17:27. doi: 10.1186/s12896-017-0348-3 (PMC5341401; doi:10.1186/s12896-017-0348-3)
Supplement: Additional file 2: — Table of T. oleaginosus homologues in TORC signaling network: Abbreviations: Trichosporon oleaginosus, TO; Schizosaccharomyces pombe, SP; Nitrogen Catabolite Repression, NCR; Saccharomyces cerevisiae, SC; Dictyostelium discodeum, DD. (DOCX 38 kb) [file 12896_2017_348_MOESM2_ESM.docx]

| **Pathway** | **Gene/Complex** | **Comment** | **TO Homologue** | **ProteinID** | **Location** |
| --- | --- | --- | --- | --- | --- |
|  |  | **TORC1** |  |  |  |
| TORC1 | *tor1* | Rapamycin binding motif present in TO | Tor2 | 284088 | scaffold_15:21383-29165 |
| TORC1 | *kog1* | Scaffold protein facilitating connection between Tor and downstream substrates | Kog1 | 284831 | scaffold_22:52969-57902 |
| TORC1 | *wat1* | In mice not essential for TORC1, but for TORC2 activity | Wat1 | 57020 | scaffold_2:285490-286968 |
| TORC1 | *tco89* | Deletion results in rapamycin hypersensitivity in SC | not found |  |  |
|  |  | **TORC2** |  |  |  |
| TORC2 | *tor2* | Only one TOR gene in TO | Tor1 |  | see above |
| TORC2 | *wat1* |  | Wat1 |  | see above |
| TORC2 | *sin1* | Conserved binding to TOR2 N-terminus | Sin1 | 69103 | scaffold_23:36948-39520 |
| TORC2 | *avo2* | Non-essential TORC2 component | not found |  |  |
| TORC2 | *ste20* | Conserved in TORC2, no common structural motifs | Ste16 | 89274 | scaffold_3:272899-277627 |
| TORC2 | *bit61* | Non-essential TORC2 component | not found |  |  |
|  |  | **SNF (AMPK) Pathway** |  |  |  |
| SNF-Path | *snf1* | Impacts on TSC1/2 | Snf1 | 283830 | scaffold_13:22589-25197 |
| SNF-Path | *LKB1* | Activation of Snf1 | not found |  |  |
| SNF-Path | *tos3* | Activation of Snf1 | Tos3 | 247203 | scaffold_38:169274-170546 |
| SNF-Path | *sak1* | Activation of Snf1 | Sak1 | 108990 | scaffold_38:167511-171260 |
| SNF-Path | *elm1* | Activation of Snf1 | Elm1 | 292545 | scaffold_38:169274-171195 |
| SNF-Path | *sip4* | Central Regulator of carbon repressed genes, activated by phosphorylated Snf1 | not found |  |  |
| SNF-Path | *reg1* | Inhibition of Snf1 | Reg1 | 264283 | scaffold_65:2237-5263 |
| SNF-Path | *glc7* |  | Glc7 | 250471 | scaffold_71:27846-28984 |
| SNF-Path | *arf3* | Activated by Snf1 in low energy environment | not found |  |  |
|  |  | **TSC Complex/TORC Upstream** |  |  |  |
| TSC PATH | *FKBP12* | Forms complex with rapamycin and inhibits TORC1 | Fkh1 | 275378 | scaffold_36:88698-89351 |
| TSC PATH | *tsc2* | Tuberin homologue | Tsc2 | 285856 | scaffold_33:86602-92917 |
| TSC PATH | *tsc1* | Hamartin homologue | Tsc1 | 315493 | scaffold_117:6411-9393 |
| TSC PATH | *Vps34* | Signals Availability of aminoacids, bypassing Tsc/RheB axis | Vps34 | 268904 | scaffold_4:261651-264613 |
| TSC PATH | *RheB* | Connects TSC-Complex to TORC1, possibly TORC2 | Rhb1 | 237912 | scaffold_71:7565-8434 |
| TSC PATH | *Erk/Rsk* | Inhibition of Tsc complex (Erk signalling) | Erk/Rsk-like | 315969 | scaffold_130:19790-24650 |
| TSC PATH | *Rtp801/L* | Activates TSc1/2, conducts signals concerning DNA damage, other stressors | not found |  |  |
| TSC PATH | *gtr1* | Make up EGO complex localized at vacuolar membrane, confers amino acid availability to TORC. Possibly bypasses Tsc1/2 Rheb axis. | Gtr1 | 300143 | scaffold_8:334087-335551 |
| TSC PATH | *gtr2* |  | Gtr2 | 238031 | scaffold_4:169222-170531 |
| TSC PATH | *ego1* |  | not found |  |  |
| TSC PATH | *ego2* |  | not found |  |  |
| TSC PATH | *ego3* |  | not found |  |  |
| TSC PATH | *npr2* | Possible TORC1 inhibitors in response to aminoacid scarcity | Npr2 | 257923 | scaffold_15:100949-103201 |
| TSC PATH | *npr3* |  | not found |  |  |
| TSC PATH | *acc1* | Acetyl-CoA carboxylase, required for initiation of fatty acid synthesis | Acc1 | 329998 | scaffold_61:23648-31248 |
|  |  | **TORC1 Downstream** |  |  |  |
| Translation | *S6k1* | Ribosomal s6 kinase. Initiation of translation and ribosome biogenesis in eukaryotes | Sch9 | 307993 | scaffold_27:135937-139531 |
| Translation | *S6* | Ribosomal Protein S6, in eukaryotes | S6b | 282616 | scaffold_5:384088-385762 |
| Translation | *4E-BP1* | Repression of translation, targets elF4E in eukaryotes | not found |  |  |
| Translation | *elF4E* | Initiation of translation, helicase, in eukaryotes | Cdc33-like | 280911 | scaffold_120:13833-15119 |
| Translation |  |  | Cdc33-like | 298705 | scaffold_1:475432-477187 |
| Metabolism | *gaf1* | Modulates sexual development in SP | not found |  |  |
| Metabolism | *PP2A* | Transcription factor, PP2A like, involved in cell cycle, stress response in SC | Ppe1 | 271546 | scaffold_13:19582-21194 |
| Lipid | *tap42* | Involved in lipid synthesis, possibly autophagy in SC | Tap42 | 240320 | scaffold_4:404467-405678 |
| Lipid | *sit4* |  | Sit4 | 298867 | scaffold_2:251612-255231 |
| Lipid | *gat1* | Transcription Activator for NCR genes. Associated with lipid replenishment in SC | Gat1 | 302500 | scaffold_31:151465-152390 |
| Lipid | *gln3* | Transcription activators associated with lipid replenishment. In SC responsible for TORC mediated response to low intracellular glutamine | not found |  |  |
| Lipid | *rtg1* |  | not found |  |  |
| Lipid | *rtg3* |  | not found |  |  |
| Transcription initiation | *elf4G* | Transcription factor, Polymerase I, in eukaryotes | Tif1 | 103441 | scaffold_36:30441-32500 |
| Transcription initiation | *maf1* | Polymerase III suppressor | not found |  |  |
| Autophagy | *atg1* | Autophagy related gene, conserved TORC substrate |  | 231999 | scaffold_43:222-3477 |
| Autophagy | *atg13* | Autophagy related gene | not found |  |  |
| Autophagy | *atg17* | Autophagy related gene | not found |  |  |
| Autophagy | *atg31* | Autophagy related gene | not found |  |  |
| Autophagy | *atg29* | Autophagy related gene | not found |  |  |
|  |  | **TORC2 Downstream** |  |  |  |
| Autophagy | *akt1/PKB* | Stress response, G1 arrest, sexual development, aa uptake, possible feedback to Tsc1/2 | Gad8 | 288963 | scaffold_97:62770-65023 |
| Autophagy | *ksg1* | Involved in cell wall integrity, activates Gad8 in SP, SC | Ksg1 | 325387 | scaffold_4:211245-215591 |
| Autophagy | *FOXO3* | Forkhead box like transcription factor, induces expression of autophagy related genes in SP | Fkh1 | 300675 | scaffold_12:188403-189818 |
| Autophagy | *atg12l* | Activation by Fkh/FoxO3, Induction of autophagy in SP, SC | Atg12 | 283501 | scaffold_10:225120-225896 |
| Autophagy | *ulk2* | Activation by Fkh/FoxO3, Induction of autophagy in SP, SC | not found |  |  |
| Cell skeleton | *RHO/RAC* | Actin organization in SP | not found |  |  |
| Cell skeleton | *PKC* | Only one PKC gene in TO, required for cell wall remodeling and sphingolipid regulation in SP | Pkc1 | 73077 | scaffold_24:189607-193675 |
| Autophagy | *PKBA* | Inhibited by Pdk1, affects actin organization, chemotaxi, cell movement in DD | not found |  |  |
|  |  | **Other** |  |  |  |
| Autophagy | *atg5* | Autophagy related gene | Atg5 | 285573 | scaffold_30:25886-27199 |
| Autophagy | *atg6* | Autophagy related gene | Atg6 | 268673 | scaffold_3:492871-494478 |
| Autophagy | *atg99* | Autophagy related gene | Atg99 | 327958 | scaffold_26:111275-114356 |
| Autophagy | *atg16* | Autophagy related gene | Atg16 | 183500 | scaffold_77:6693-7765 |
