# Supplementary figures and images for: The effects of TORC signal interference on lipogenesis in the oleaginous yeast Trichosporon oleaginosus
Source: BMC Biotechnol. 2017 Mar 7;17:27. doi: 10.1186/s12896-017-0348-3 (PMC5341401; doi:10.1186/s12896-017-0348-3)

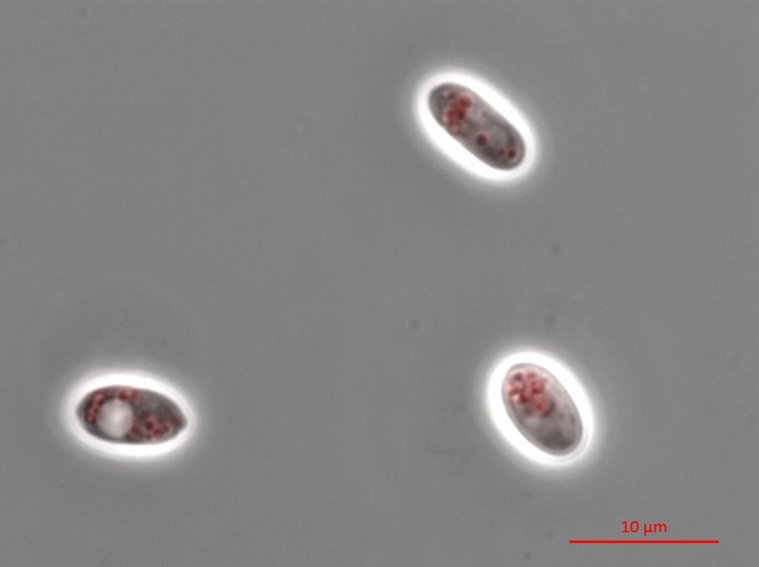

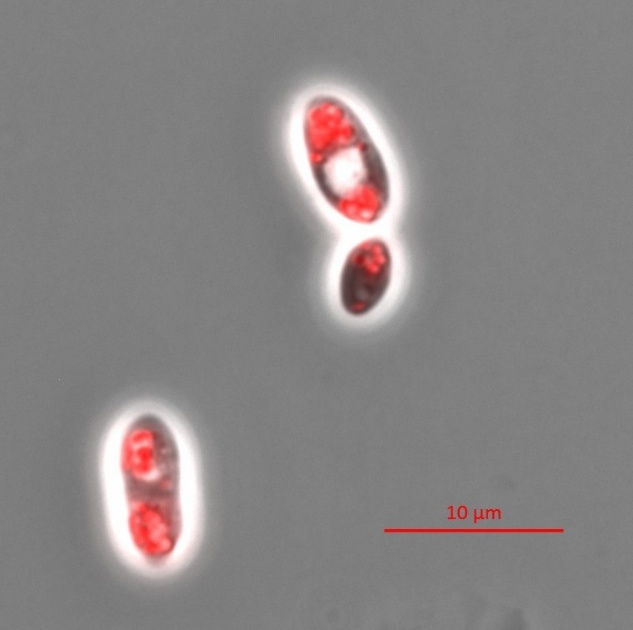


With Rapamycin Without Rapamycin

Supplement: Additional file 3: — Fluorescence microscopy: 200 μL T. oleaginosus cells grown for 72 h in YPD with and without 5 μM rapamycin supplementation were pelleted, washed with ddH2O and resuspended in the same amount of water. 25 μL DMSO and 25 μL nile red (50 mg/ml) in DMSO were added and incubated in darkness for 10 min. Images were taken on a Zeiss Axio Lab A1 with an Axio Cam ICm1 (Oberkochen, Germany). Fluorescence was measured with a 525/25 filter with an exposure time of 500 ms. (DOCX 154 kb) [file 12896_2017_348_MOESM3_ESM.docx]
