# Supplementary material for: The effects of TORC signal interference on lipogenesis in the oleaginous yeast Trichosporon oleaginosus
Source: BMC Biotechnol. 2017 Mar 7;17:27. doi: 10.1186/s12896-017-0348-3 (PMC5341401; doi:10.1186/s12896-017-0348-3)
Supplement: Supplementary file 3 [file 12896_2017_348_MOESM1_ESM.docx]

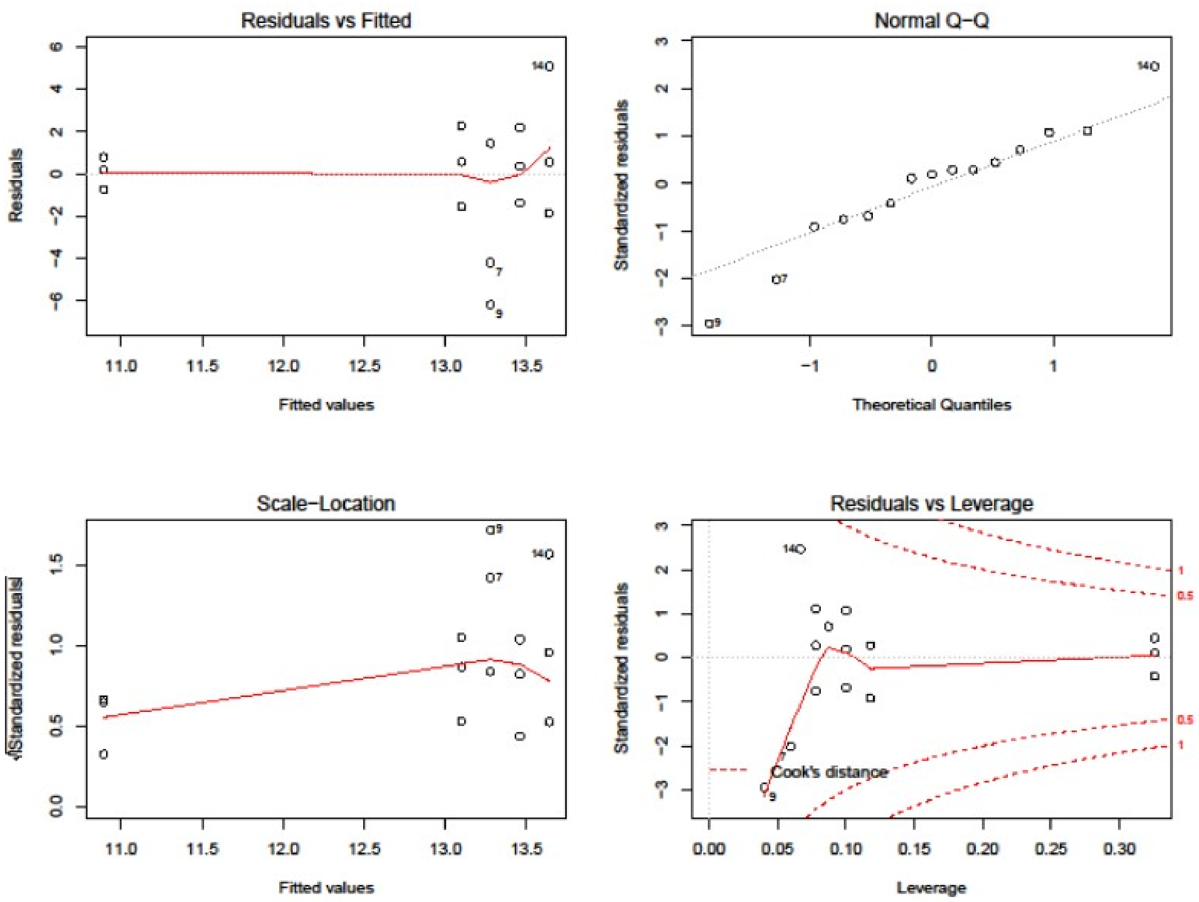

Residuals vs Fitted
Residuals
Fitted values
14
7
9
Normal Q−Q
Standardized residuals
Theoretical Quantiles
14
7
9
Scale−Location
√|Standardized residuals|
Fitted values
9
14
7
Residuals vs Leverage
Standardized residuals
Leverage
14
9
Cook's distance
1
0.5
0.5
1
